# Management of Patients With Skin Cancers: Basal Cell Carcinoma and Melanoma

**DOI:** 10.6004/jadpro.2017.8.3.5

**Published:** 2017-04-01

**Authors:** Brianna Hoffner, Daniel M. Siegel

**Affiliations:** 1 University of Colorado Cancer Center, Denver, Colorado;; 2 SUNY Downstate Medical Center, Brooklyn, New York

## Abstract

Novel therapies are changing the treatment paradigm for both melanoma and advanced/metastatic basal cell carcinoma. While immunotherapies are increasing survival benefits in melanoma, they are associated with unique immune-related adverse events.

Recent advances in understanding of the molecular biology and genetics of advanced basal cell carcinoma (BCC) and melanoma have helped to expand treatment options for both conditions and improve outcomes for patients. In the process, the advances have increased the informational and clinical knowledge demands of physicians and introduced new roles and responsibilities for advanced practitioners.

The changing clinical landscape of advanced BCC and melanoma was discussed at JADPRO Live 2016 by Brianna Hoffner, MS, ANP-BC, AOCNP®, of the University of Colorado Cancer Center, Denver, and Daniel M. Siegel, MD, MS, FAAD, FACMS, of SUNY Downstate Medical Center in Brooklyn, New York.

## BASAL CELL CARCINOMA

Advanced BCC represents a new addition to most practitioners’ medical vocabulary, a result of the development of nonsurgical options for the disease, said Dr. Siegel. He noted that BCCs once were described in less-than-scientific, though more colorful, terms and were considered challenging but manageable by surgeons.

"These are the sort of biggies that I used to look at and say, ’I can do it. I’m the fellowship-trained Mohs surgeon with 30 years of experience under my belt.’ But was that the best thing for the patient?" he commented.

The advent of hedgehog pathway inhibitors, such as vismodegib (Erivedge), introduced patients and clinical practitioners to pharmacologic options for high-risk advanced BCC, as defined by size, location, depth of invasion, and related factors (National Comprehensive Cancer Network [NCCN], 2016). If a lesion is resectable, even at recurrence, surgery remains the best option, possibly followed by radiation therapy, depending on the margin status, extent of perineural invasion, and other factors.

After primary treatment, patients should be followed for recurrence at 6- to 12-month intervals. Local recurrence can be retreated surgically, with or without radiation therapy. Metastatic recurrence also can be treated surgically, but increasingly, hedgehog inhibitors are being used in this case.

Prior to the introduction of the first hedgehog inhibitor, several topical and systemic therapies were available for BCC. Systemic therapies, such as cisplatin plus fluorouracil (5-FU) produced mediocre results, at best, admitted Dr. Siegel. Use of topical agents was limited to low-risk BCC lesions. Options included 5-FU, imiquimod, photodynamic therapy, and cryotherapy (NCCN, 2016).

These treatments resulted in a 5-year disease-free survival of about 84% in superficial BCC. The remaining patients developed recurrences, sometimes multiple recurrences, he noted.

The scientific route to hedgehog pathway inhibitors began with laboratory studies of fruit flies. Nobel Prize–winning research led to the discovery of mutations in genes that control the development of the segmented anteroposterior body axis of the fly, identification of a group of genes involved in the development of body segmentation, and subsequently to identification of the Drosophila hedgehog gene as a key controller of differentiation between anterior and posterior parts of individual body segments.

Most BCC lesions arise from alterations in the hedgehog-signaling pathway. In most cases, the alterations lead to loss of function of patched homologue 1 (PTCH1), which normally inhibits smoothened homologue (SMO)-signaling activity (Sekulic et al., 2012).

When the hedgehog ligand binds to PTCH1, SMO migrates to the cell membrane, causing signal transduction and target gene expression, said Dr. Siegel. An oral hedgehog pathway inhibitor binds to SMO to inhibit signal transduction and target-gene expression. Vismodegib became available in 2012, and the US Food and Drug Administration (FDA)-approved sonidegib (Odomzo) in 2015.

"The hedgehog pathway inhibitors made it to market on the basis of results from phase II studies. The results were so good that the government did not insist on phase III studies," said Dr. Siegel. "We observed some pretty dramatic results (see Figure). Nothing had been doing that up to that point."

**Figure 1:**
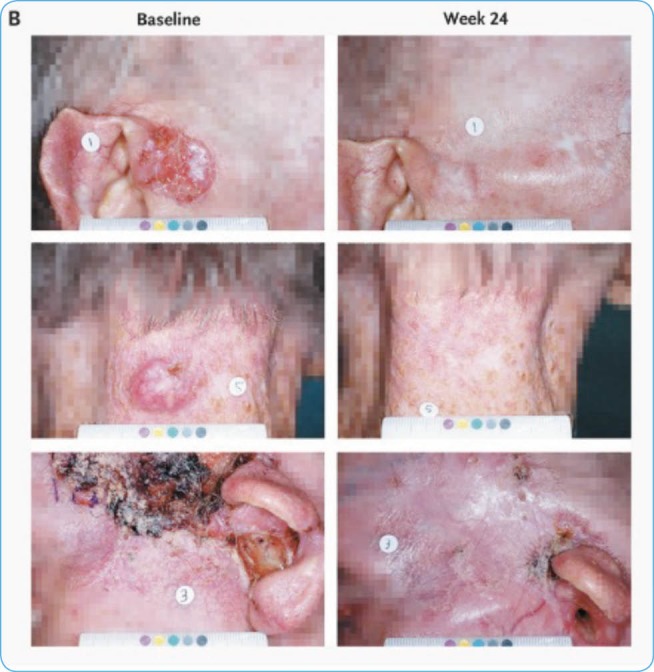
Basal cell carcinoma at baseline (left) and 24 weeks after treatment with vismodegib (right). Adapted from Sekulic et al. (2012).

Several adverse events occur commonly with hedgehog inhibitors, including muscle spasms, alopecia, dysgeusia, decrease in weight, fatigue, nausea, decreased appetite, and diarrhea (Sekulic et al., 2012). Less commonly, patients develop amenorrhea, hyponatremia, hypokalemia, and azotemia. Most side effects are mild or moderate, and grade 3/4 adverse events are uncommon. Patient follow-up does not include any specific laboratory tests for patients treated with vismodegib, whereas prescribing information for sonidegib does recommend a few tests, said Dr. Siegel.

Other hedgehog inhibitors are in various stages of clinical development, and some are being evaluated in other types of cancer, including chronic myeloid leukemia, acute myeloid leukemia, small cell lung cancer, and ovarian cancer.

**Locally Advanced or Metastatic Disease**

Recent studies of approved and investigational hedgehog inhibitors have added to the understanding of how to use the drugs and provided new insights into their clinical use. A trial of vismodegib in 1,215 patients with locally advanced or metastatic BCC confirmed the original clinical trial results in a "real-world" setting (Hansson et al., 2016).

A study employing deep-sequencing analysis of metastatic cutaneous BCC and squamous cell carcinoma (SCC) identified distinctive genomic profiles for the diseases and suggested new routes for targeted therapies (Ross et al., 2016). Both types of skin cancer had high mutational loads, said Dr. Siegel. Metastatic SCC exhibited more cell-cycle dysregulation, whereas metastatic BCC had more sonic hedgehog pathway alterations, including PTCH1 and SMO genetic alterations associated with vismodegib resistance.

Analysis of 30-month follow-up data from a trial of sonidegib showed better tolerance with the approved dose of 200 mg compared with 800 mg daily, as well as a higher objective response rate with the approved dose (56% vs. 45%). The drug had similar activity in patients with aggressive and nonaggressive BCC (Dummer et al., 2016).

French investigators reported data on resistance in 207 vismodegib-treated patients with locally advanced BCC (Basset-Seguin et al., 2016). The results showed a 4.7% rate of primary resistance, and 8.7% of patients developed secondary resistance.

## MELANOMA

In contrast to most other malignancies, the incidence of melanoma continues to increase, especially among younger women. Use of tanning beds is a possible contributor to the rising incidence of melanoma, which likely is multifactorial, said Ms. Hoffner. The 5-year survival is 91.5% overall, but it falls off dramatically with metastatic disease.

Melanoma offers multiple targets for treatment and has proved to be amenable to immunotherapies as well (Batus et al., 2013). Oncolytic immunotherapies, immune checkpoint inhibitors, and cytokines have been evaluated in melanoma, as have targeted therapies from the BRAF and MEK inhibitor classes (including combinations).

**Talimogene Laherparepvec**

An oncolytic immune therapy that includes an attenuated live herpetic virus, talimogene laherparepvec (T-VEC; Imlygic) received FDA approval in late 2015 and is administered directly into melanoma lesions. Upon injection into a lesion, the agent induces tumor lysis by means of T-cell activation, leading to a systemic immune response (Pol, Rességuier, & Lichty, 2014; Hawkins, Lemoine, & Kirin, 2002; Mullen & Tanabe, 2002; Fukuhara & Todo, 2007).

"This is important for advanced practice providers to know about. Many institutions are trying to figure out the best way to administer the treatment, and advanced practice providers often are best positioned to do that," Ms. Hoffner said.

**Immunotherapy**

The field of immunotherapy has expanded greatly over the past 5 years. Prior to 2011, the only approved immunotherapy for melanoma was high-dose interleukin-2, approved in 1992. Beginning with ipilimumab (Yervoy) in 2011, the FDA approved five additional immunotherapeutic agents for melanoma indications: pembrolizumab (Keytruda; 2014), nivolumab (Opdivo; 2014), ipilimumab-nivolumab combination therapy (2015), and T-VEC (2015).

Ipilimumab, an inhibitor of cytotoxic T-lymphocyte–associated protein 4 (CTLA-4), provided the first demonstration that immunotherapy could offer outcomes superior to those achieved with conventional chemotherapy for melanoma (Robert et al., 2011). Follow-up in the landmark trial showed that ipilimumab could achieve durable responses associated with improved long-term survival (Maio et al., 2015).

A pivotal trial comparing nivolumab, an inhibitor of the receptor for programmed cell death protein 1 (PD-1), and dacarbazine in previously untreated advanced melanoma yielded even more impressive results, demonstrating a 58% reduction in the survival hazard in favor of the immune checkpoint inhibitor (Robert et al., 2015a). Shortly, thereafter, pembrolizumab demonstrated superior progression-free survival (PFS) in a randomized comparison against ipilimumab (Robert et al., 2015b).

The success of individual immunotherapeutic agents in melanoma subsequently led to evaluation of combination therapy. Another landmark trial demonstrated that the combination of ipilimumab and nivolumab significantly improved PFS as compared with either agent alone (Larkin et al., 2015).

Immunotherapeutic agents have different adverse-event profiles, said Ms. Hoffner. Anti–CTLA-4 therapy is associated with higher rates of diarrhea and colitis, which can be severe in some cases (including bowel perforation, sepsis, and death). Severe colitis also occurs more commonly with combination therapy as compared with a PD-1 inhibitor alone.

Hepatotoxicity, including hepatitis, occurs in 2% to 9% of patients treated with ipilimumab, as compared with < 1% with the PD-1 inhibitor pembrolizumab. Combination therapy, including the combination of an immunotherapeutic agent with chemotherapy or targeted therapies, also has been associated with more hepatotoxicity, said Ms. Hoffner.

Dermatitis occurs in as many as 40% of patients treated with anti–CTLA-4 therapy and in about 30% of patients treated with a PD-1 inhibitor, she continued. The rate is higher among patients who receive combination therapy.

Various autoimmune endocrinopathies have been reported in patients treated with immunotherapeutic agents. Hypophysitis, thyroid disease, and abnormal thyroid function tests have all been reported and occur more often in patients who receive combination therapy. The underlying mechanisms of the endocrinopathies remain unclear, said Ms. Hoffner.

Immunotherapy is associated with a long list of other adverse events, which occur with varying frequency. They include ocular, neurologic, and pulmonary toxicities; sarcoidosis; systemic vasculitis; autoimmune pancreatitis; and hematologic disorders. General guidelines for managing the adverse effects have evolved with clinical experience, said Ms. Hoffner. The first step in each case is to rule out all other potential causes of an adverse event (Table).

**Table:**
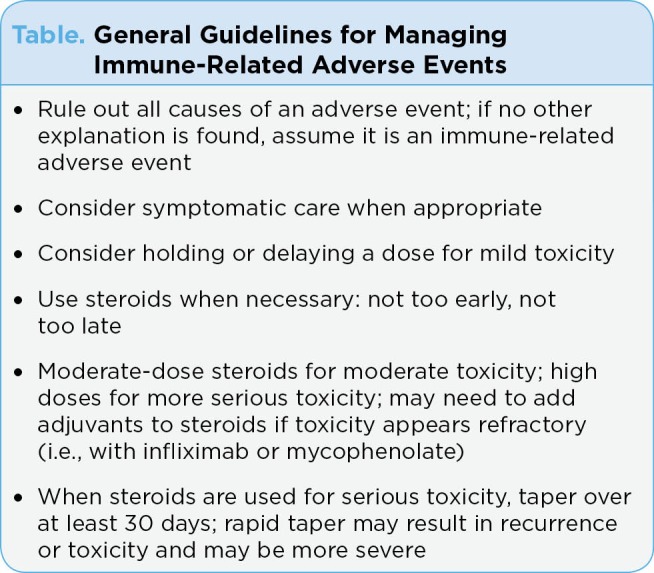
General Guidelines for Managing Immune-Related Adverse Events

**Targeted Therapies**

About half of all melanomas are associated with the *BRAF* V600E mutation. Development of the first drug targeting that mutation had a transformative effect on management of patients with advanced melanoma. A landmark randomized trial showed response rates of 48.4% with the BRAF inhibitor vemurafenib, compared with 5.5% with dacarbazine. The dramatic difference in response rate translated into significant improvement in both PFS and overall survival (Chapman et al., 2011).

However, responses to vemurafenib often proved to be short-lived, as acquired resistance emerged in most patients, said Ms. Hoffner. As with the immunotherapeutic drugs, interest in combination therapy soon led to randomized trials of targeted agents. Similar to the experience with immunotherapy combinations, a randomized trial demonstrated superior response rate, response duration, and overall survival with the combination of a BRAF inhibitor and a MEK inhibitor as compared with a BRAF inhibitor alone (Robert et al., 2015c).

"Acquired resistance to BRAF therapy generally occurs at about 6 months," stated Ms. Hoffner. "BRAF and MEK inhibitors are approved in patients with *BRAF* V600 mutations."

Adverse events associated with BRAF inhibition include several types of dermatologic toxicities, including potentially severe photosensitivity, secondary squamous cell carcinomas, keratoacanthomas, and rash. Other potential adverse effects include uveitis, QTc prolongation, and hepatotoxicity, as well as more general adverse events such as alopecia, arthritis, nausea, and fatigue.

MEK inhibitors have less single-agent activity, as compared with BRAF inhibitors (objective response rate of about 25%). As a result, the agents are almost always used in combination therapy, said Ms. Hoffner. Adverse effects associated with MEK inhibitors are similar to those seen with BRAF inhibitors.

Phase I/II clinical trial data showed that the combination of dabrafenib (BRAF inhibitor) and trametinib (MEK inhibitor) resulted in an overall response rate of 76%, compared with 54% for single-agent dabrafenib (Flaherty et al., 2012). The FDA approved the combination for melanoma in January 2014.

"The BRAF and MEK inhibitor combinations work better and last longer, and the side-effect profile is better," said Ms. Hoffner.
